# Examining the Associations between Indigenous Rangers, Culture and Wellbeing in Australia, 2018–2020

**DOI:** 10.3390/ijerph18063053

**Published:** 2021-03-16

**Authors:** Alyson Wright, Mandy Yap, Roxanne Jones, Alice Richardson, Vanessa Davis, Raymond Lovett

**Affiliations:** 1 National Centre for Epidemiology and Population Health, Research School of Population Health, The Australian National University, 64 Mills Road, Acton 2600, Australia; roxanne.jones@anu.edu.au (R.J.); raymond.lovett@anu.edu.au (R.L.); 2 Centre for Aboriginal Economic and Policy Research, The Australian National University, Acton 2600, Australia; mandy.yap@anu.edu.au; 3 Statistical Consulting Unit, The Australian National University, Acton 2600, Australia; alice.richardson@anu.edu.au; 4 Tangentyere Council, 1 Elders St, Alice Springs 0870, Australia; vanessa.davis@tangentyere.org.au

**Keywords:** survey, Indigenous, rangers, land management, language, country, environment, health and wellbeing, Aboriginal and Torres Strait Islander

## Abstract

The centrality of culture to Indigenous peoples’ health and wellbeing is becoming increasingly acknowledged in government policy. In Australia, the Indigenous Ranger program is a leading example of employment that supports increased cultural participation. In 2017, we demonstrated higher life satisfaction and family wellbeing among Indigenous Rangers compared to non-Rangers in Central Australia. Using an expanded national dataset, this present study aimed to: examine if associations between Ranger status and wellbeing continued to be observed in Central Australia; assess if these associations were observed among non-Central Australian Rangers; and, quantify the effect of mediating variables (Rangers status, cultural factors) on wellbeing outcomes. We analyzed Mayi Kuwayu baseline data (*n* = 9691 Aboriginal and Torres Strait Islander people) and compared participants who identified as past or currently employed Rangers compared to non-Rangers across two geographic locations (Central Australia, non-Central Australia). Ranger participation was significantly associated with very high life satisfaction and family wellbeing in Central Australia (high life satisfaction PR 1.31, 95% CI 1.09–1.57, and family wellbeing (PR 1.17, 95% CI 1.01–1.36) and non-Central Australia (high life satisfaction PR 1.29, 95% CI 1.06–1.57), family wellbeing (PR 1.37, 95% CI 1.14–1.65). These findings concord with those observed in the 2017 proof-of-concept study. Additionally, we found that Ranger status partially mediated the relationships between existing cultural practices (first language as your Indigenous language and living on your country) and the two wellbeing outcomes. Current cultural practices, spending time on country and speaking your Aboriginal language, also partially mediated the associations between Ranger status and high life satisfaction, and between Ranger status and high family wellbeing. This analysis supports evidence that both Ranger employment and cultural participation are contributors to wellbeing. Ranger work is not only good for land, but it is good for people. As such, determining policies that mutually acknowledge and enhance culture, health and wellbeing will likely have additional benefits for the broader Aboriginal and Torres Strait Islander population.

## 1. Introduction

The important contribution of culture for Indigenous people’s health and wellbeing is increasingly acknowledged [1]. Earlier studies have identified positive associations between participation in cultural activities, including through caring for and living on land (country) [2,3,4], speaking Indigenous language [5,6,7,8], passing on knowledge and beliefs, [9] and, practicing local customs [10,11], and the wellbeing of Indigenous people within Australia and internationally. These studies have a dual purpose: to gain recognition of the contribution of culture for Indigenous peoples’ health and wellbeing; and, to shift policy conversations from a deficit discourse to a strength-based dialogue, focusing on what is working rather than not working. In doing so it asks of government and those working to improve health outcomes of Indigenous peoples [12], to establish policies and programs that mutually enable health, wellbeing and culture to flourish.

For many Aboriginal and Torres Strait Islander peoples in Australia, culture is central to their life and fundamental for wellbeing [13]. Parter et al. (2019) asserted that “health for Indigenous people includes the right to practice their cultural values, beliefs and norms including such practices that reaffirm connection to Country, languages…” [14]. In this paper, we adopt a broad definition of culture as a system of shared beliefs, values, customs and behaviours that are used by members of a group to shape their worldview and understanding of themselves and others [15]. Culture is not something possessed by the ‘other’ such as a specific value or physical appearance but rather it is a set of values [16]. In line with this and supported in United Nations declaration of the rights of Indigenous people, being “Indigenous” is based on a person identifying as Indigenous (or in Australia as Aboriginal and/or Torres Strait Islander) rather than defining what it is to be Indigenous [17]. Culture can encompass aspects of Aboriginal and Torres Strait Islander peoples’ distinct languages, connection to country, knowledge and beliefs, self-determination and leadership, cultural expression and continuity, and, family, kinship and community [18].

The importance of Aboriginal and Torres Strait Islander culture is increasingly recognised as central in Indigenous policy frameworks [19,20], but how these tenets support culture is still emerging [14]. Arguably, the leading example of culture being operationalised in Indigenous development programs in Australia is the employment of Indigenous Rangers. Established with the Community Development Employment Program (CDEP) initiatives in the early 80 s, Indigenous Rangers have continued to offer Aboriginal and Torres Strait Islander people work on country, that utilises cultural and environmental knowledge to manage land. From 2007/08, funding for Indigenous Ranger transitioned to the Australian Government’s Working on Country program with the abolition of CDEP. The Working On Country program currently supports over 100 Ranger groups nationally, who undertake culturally-informed Indigenous conservation and land management activities [21]. In 2009, Garnett et al. were critical of the Working on Country program stating that program funding was largely derived from environmental budgets and outcomes of the program were fundamentally environmental objectives [22]. These researchers argued that the benefits of being involved in Indigenous conservation and land management initiatives are intersectional, across multiple policy domains, creating an opportunity for diversifying potential program investments.

More recently, a study of Aboriginal and Torres Strait participants involved in land and sea management programs from communities in far North Queensland (Australia) reported the value of the land and sea management programs were not only environmental, but importantly, encouraged the use of cultural practices, leading to an increase in cultural and social benefits for the community [23]. These papers raise the question—do activities on country facilitate the building and reinforcement of cultural involvement and therefore wellbeing and in what ways? Being able to measure the multiple benefits derived from being employed as a Ranger, including the health and wellbeing outcomes, not only contributes to evidence that supports diversifying of funding bases for Ranger programs, but also adds to important evidence on the relationship between culture and wellbeing.

### The Mayi Kuwayu Study and Proof-of-Concept Study

Mayi Kuwayu: The National Study of Aboriginal and Torres Strait Islander Wellbeing has been designed to provide national data on culture, health and wellbeing [24]. As part of the Mayi Kuwayu survey development, a proof-of-concept study was undertaken, examining the associations between participation in a Ranger program and health and wellbeing outcomes among participants of Central Australia. This 2017 proof-of-concept study demonstrated higher prevalence of wellbeing outcomes for Rangers compared to non-Rangers [25]. We defined wellbeing using both subjective measures (such as psychological wellbeing, life satisfaction and general health) and culturally specific measures (including family wellbeing). The findings established that Rangers compared to non-Rangers reported higher levels of family wellbeing and life satisfaction. Results for general health were also of the same magnitude and direction, but not statistically significant. Psychological wellbeing was not different between Ranger and non-Rangers. We hypothesized that these family wellbeing and life satisfaction benefits could partially be explained by the increase in cultural activities that Rangers do as part of their work.

Studies on culture and wellbeing, including the proof-of-concept study described above, have been criticized because they are small scale [1] and often use proxy indicators of culture, rather than direct measures. In this current paper, we use direct measures of culture to better understand the relationships between Ranger and wellbeing outcomes. We also expand on the 2017 Ranger study, by examining if the associations continue in the Central Australian population and whether they are observed in participants outside of Central Australia. Using the second release of baseline data from the Mayi Kuwayu Study (*n* = 9691) we aim to:

1. examine if associations between Ranger status and health and wellbeing continue to be observed in Central Australia;
2. examine if the associations between Ranger status and health and wellbeing are observed among non-Central Australian Rangers; and,
3. determine the mediating effect of Indigenous cultural factors and Ranger status on wellbeing outcomes.

## 2. Methods

### 2.1. Mayi Kuwayu Study

Mayi Kuwayu is open to participation from all Aboriginal and Torres Strait Islander adults over the age 16 years. Details of the study have been described elsewhere, but importantly this study collects data via surveys asking participants to self-report on questions regarding culture, wellbeing and health, and key socioeconomic characteristics [24]. The Aboriginal-led study was developed following initial proof-of-concept studies, including the first analysis of Rangers wellbeing. The Mayi Kuwayu Study commenced baseline recruitment in May 2018, employing the use of both postal and field-based recruitment methods [26]. Recruitment for the Study is ongoing and the analysis in this paper used the second release of baseline data (CW R2.0). A comparison of elements of the 2017 proof-of-concept study [25] and this current study are listed in Table 1.

### 2.2. Participant Eligibility

A participant was eligible for the inclusion in this analysis if they responded to the Mayi Kuwayu survey between May 2018 to April 2020 and were included in the Mayi Kuwayu dataset (CW R2.0). Rangers were defined as those who selected the option “Ranger program” to the question “Have you ever participated in any of these programs?”. “Ranger program” was one option among a list of 12 programs. Non-Rangers were any Mayi Kuwayu participant in this dataset who did not select the ”Ranger program” option. In the analysis, references to Rangers included anyone who was formerly or is currently a Ranger irrespective of their time of involvement with Ranger work.

To enable analysis and comparison between Central Australia and non-Central Australia, information from participant responses on postcode was recoded to create a binary location variable. This variable included two categories, the first category was defined as participants in “Central Australia” and included participants with postcodes listed in Supplementary Material Table S2. The Central Australian region used in the analysis crosses state boundaries in order to capture the tri-border area of South Australia, Northern Territory and Western Australia, and broader desert area. This tri-border region has related Aboriginal language groups and close affiliations. The second category was “non-Central Australia” and included participants from the remainder of the cohort.

### 2.3. Variables

#### 2.3.1. Wellbeing Outcome Measures

Life satisfaction was measured according to responses to the question, “How satisfied are you with your life?”, with response options “a lot”, “a fair bit”, “a little bit” and “not at all”. These were then categorized as low to high life satisfaction (a little bit, not at all, a fair bit) or very high life satisfaction (a lot).

General health was measured according to the question “How would you rate your general health?”; response options were “poor”, “fair”, “good”, “very good” or “excellent” [27]. Responses were categorized into two groups: poor to fair health (poor and fair) and very good health (good, very good or excellent).

Psychological wellbeing was measured using the Kessler Psychological Distress (K5) scale [28]. Responses to the questions were summed; participants were categorized as having low/moderate (score 5–11) or high/very high levels of distress (score 12–25). Scores were only calculated for participants with complete data on the five items. For the analysis, those with “low/moderate distress” were defined as having high psychological wellbeing; those with “high/very high distress” were defined as having low/moderate psychological wellbeing.

Family wellbeing was measured using a modified version of the Western Australian Aboriginal Child Health Survey family functioning scale [29]. It is measured according to responses to a set of nine questions each with response options of “not at all” (1), “a little bit” (2), “a fair bit” (3) to “a lot” (4), and “unsure” (0). Responses were summed (range: 0–36), and participants were categorized as having low/moderate (score 0–29) or high family wellbeing (score 30–36). Responses to the nine questions were summed for participants with complete data only; participants missing responses to any of the questions were coded as missing. All outcome measures above were coded as binary to enable the regression analysis.

#### 2.3.2. Covariates

Socioeconomic characteristics: Gender was coded as female, male or missing. Due to the small number of participants selecting “other” gender (*n* ≤ 5), these were recoded as missing. Age was recoded into three age groups: 16–34 years, 35–54 years, and 55 years and older, and was based on the groupings applied in the proof-of-concept paper. Highest attained education qualification was categorised into three groups: Year 10 or below (no school, primary school and intermediate certificate); Year 12 or trade or certificate (higher school, diploma/certificate, trade); and, university qualifications.

Participants were categorized as employed if they reported working part or full-time or if they were studying (studying was included in employment as these participants had a similar profile across other socioeconomic variables to those employed); and categorized as not employed if they were not working (including being retired, on a pension or carer). Family financial status was derived from the question, “which words best describe your family’s money situation?” Responses were recoded: high (included participants who selected “we have a lot of savings”), medium (included participants who selected “we have some savings” or “we have just enough to get us to the next payday”), low (included participants who selected “we run out of money before payday” or “we are spending more than we get”), and missing (included responses of unsure or missing). Remoteness was coded by the Mayi Kuwayu data managers as per the Australian Statistical Geographical Standard which includes major city, inner regional, outer regional, remote and very remote [30].

Health characteristics: Health characteristics included a few health conditions and health risk factors reported by participants. A derived health condition score was created from three self-reported questions asking whether the participants had been informed by a doctor that they had diagnoses of heart disease, diabetes or stroke. For the analysis participants were initially coded as 1 “ever having a condition” or 0 “never having each condition”. This was summed for a composite health condition score of the number of conditions (range: 0–3). Participants were categorised as having no conditions or 1 or more health conditions.

A derived health risk score was created from participants reporting a doctor had informed them that they had diagnoses of high blood pressure, high cholesterol or self-reported being a current smoker. A composite health risk score that summed the number of risks reported (range: 0–3) was first created. Participants were then categorised as having “no health risk factors” (0) or “one or more of the health risk factors” (1). The health risk factor score was coded as missing if participants were missing data on smoking status.

#### 2.3.3. Cultural Factors

To expand on the 2017 proof-of-concept study, we considered several measures of culture (Indigenous language use and connection to country) as mediators for associations between Ranger status and wellbeing outcomes and Ranger status as meditator between cultural measures and wellbeing. Several questions related to language and connection to country were used in the analysis representing a number of aspects which Indigenous peoples and communities have expressed as being important to their sense of wellbeing [13,18,31]. Two variables in language and connection to country are assumed to precede Ranger status (first language and living on country) while others (speaking language, confidence in speaking language, cultural responsibility, time on country) are assumed to follow Ranger status. In addition, the questions explore different constructs (temporal, current use and participation) of language and connection to country measures.

Language: Three different language questions were considered in the analysis. Firstly, we included “What is your first language?”. Responses were coded as 1 for Aboriginal or Torres Strait Islander language or 0 (includes English or other). For the mediation analysis using this variable, Ranger status was considered the mediator and language, the exposure. Secondly, we looked at language measures that could be considered as mediators, which included “Do you speak any Aboriginal/Torres Strait Islander words or languages?”. Responses to this question included “want to but can’t”, “not at all”, “a little bit”, “a fair bit”, and “a lot”. We also considered “I am confident in speaking words or language” as a mediator, which had similar categorical responses as the question listed above.

Connection to country: Three questions were also considered for connection to country. The question: “do you live on your country?” was used as an exposure and Ranger status as meditator in the mediation analysis. Responses were coded 1 if response was “yes” or 0 if “no” or “unsure”. When we analysed connection to country as mediator, we used the following questions “Do you have special cultural responsibilities for country?”. A responses was coded as 1 if participants responded “yes” (including “yes mothers side”, “yes fathers side”, or “yes other country”) and 0 if participants responded “no” or “unsure”. Lastly, the question of “How much time do you spend on country?” was used as mediator and categories for response included “want to but can’t”, “not at all”, “a little bit”, “a fair bit”, and “a lot”.

### 2.4. Statistical Analysis

We conducted a descriptive analysis of the demographic factors, health conditions and health risk factors, and wellbeing outcomes for the Ranger and non-Ranger groups in the total sample and by location separately. We used log-binomial models to calculate prevalence ratios (PRs) and 95% Confidence Intervals (CIs) for each outcome (life satisfaction, general health, psychological wellbeing and family wellbeing) for Rangers compared to non-Rangers in Central Australia and non-Central Australia, separately. In keeping with the method deployed in the proof-of-concept analysis [25], log-binomial regression models were used for the univariate and multivariate analysis, as this regression model was preferred over logistic regression because the outcomes of interest in the study population were common [32]. All models excluded participants missing data on the outcome of interest.

To test if key demographic and health factors accounted for the differences in wellbeing outcomes between Rangers compared to non-Rangers, we repeated the regression analysis controlling for age and gender across the models and separately adjusting for: education, employment status, financial status, health condition score, health risk factor score and remoteness (remoteness was not adjusted for in Central Australian group because all participants reside in remote or very remote areas). We tested the sensitivity of results in the sample by comparing results in the Central Australian group to results in the non-Central Australian group.

Participants with missing data on the covariates were included as a separate missing category; as such, the total sample size was consistent for all models with the same outcome. Where a cell of the control variables contained a count of zero, we did not include a missing category in the regression. Stata 16 was used for all analysis.

#### Mediation Analysis

We conducted a mediation analysis to assess the extent to which culture variables or Ranger status mediated the relationships between exposures and the wellbeing outcomes. We ran a counter-factual mediation analysis using Stata PARAMED (StataCorp, Texas, United States of America) which compares two regression models: the first model regresses the outcome (a. family wellbeing, or b. life satisfaction) on the main exposure (a. Ranger status, b. first language, or c. lives on country), the proposed mediator (a. speaks Aboriginal or Torres Strait language, b. spends a lot of time on country, or c. Ranger status) and specified covariates; the second model regresses the proposed mediator on the exposure variable and covariates.

The mediation analysis was carried out using log binomial regression in keeping with the methods deployed above and because outcomes were common [32]. We dichotomized the categorical language and connection to country variables using linear binary cut points. The grouping for the cut-points were:

- **≥a little bit** included the groupings “a little bit, a fair bit, a lot” (1) and “want to but can’t, not at all” (0);
- **≥a fair bit** included the groupings “a fair bit, a lot” (1) and “want to but can’t, not at all, a little bit” (0); and,
- **a lot** included the groupings “a lot” (1) and “want to but can’t, not at all, a little bit, a fair bit” (0).

The mediation adjusted for covariates, age and gender, and an exposure mediator interaction to account for any interaction effect between the cultural measures and Ranger status.

We estimated the natural direct effect and the natural indirect effect on outcomes of family wellbeing and life satisfaction. The total effect was derived from the product of the natural direct and indirect effect. We divided the logarithm of the indirect effect by the logarithm of the total effect to determine the proportion of the total association that was mediated, as per the equation below:

% Mediated = ln(indirect effect)/ln(total effect)

### 2.5. Ethics and Data Use Approval

Ethics approval for the conduct of the Mayi Kuwayu Study was granted by state and territory jurisdictions (as listed in the ethics section). The analysis in this paper was granted ethics approval by Central Australia Human Research Ethics Committee (19-3315) and the Australian National University (2019/129).

The use of Mayi Kuwayu data is governed by an independent group of Aboriginal and Torres Strait Islander leaders, the Mayi Kuwayu Data Governance Committee, who assessed the use of the current study’s data against the Maiam nayri Wingara Indigenous Data Sovereignty Principles. Data use was granted by the Mayi Kuwayu Data Governance Committee in November 2019, the committee also reviewed and approved the findings prior to publication. In development of this paper and the analysis we worked closely with Indigenous Ranger groups, including the Central Land Council Rangers, and with Aboriginal and Torres Strait Islander researchers in the Mayi Kuwayu Study. Aboriginal researchers who authored this paper include: RJ (Palawa); VD (Western Arrernte); and, RL (Wongaibon).

## 3. Results

### 3.1. Participant Characteristics

#### Socioeconomic

There was a total of 9691 eligible Mayi Kuwayu participants included in this analysis. The Central Australian participants included 102 Rangers and 494 non-Rangers, whilst non-Central Australian participants included 164 Rangers and 8931 non-Rangers (Table 2). Among the total cohort, Rangers compared to non-Rangers were younger (27.8% of Rangers were ≥55 years compared to 39.2% of non-Rangers), included more males (62.8% of Rangers were male compared to 37.2% of non-Rangers), were more likely to live in very remote areas (37.2% of Rangers lived in very remote areas compared to 5.8% of non-Rangers), and have higher employment levels (63.9% of Rangers were employed compared to 52.9% of non-Rangers). There were higher proportions of Rangers who reported being employed in Central Australia (80.4%) compared with: non-Rangers in Central Australia (43.1%); non-Rangers (53.4%) not in Central Australia; and, Rangers (53.7%) not in Central Australia (Table 2).

Financial status and education levels were similar between Rangers and non-Rangers across the sample. Participants in Central Australia were only from regional, remote and very remote areas, but participants outside Central Australia were across all remoteness areas. Further information on health conditions and risk factors of the participants is presented in Supplementary Material Table S3.

### 3.2. Cultural Factors

More Rangers reported knowing and using their Aboriginal or Torres Strait Islander language than non-Rangers (see Table 3), with higher proportions of participants who spoke and used their language in Central Australia. For example, 61.8% of Rangers and 56.1% of non-Rangers first language was their Aboriginal or Torres Strait Islander language in Central Australia. For the non-Central Australian group, 10.4% of Rangers and 3.7% of non-Rangers used their Aboriginal or Torres Strait Islander language.

In Central Australia, 65.8% Rangers reported spending a lot of time on country and 67.3% reported having cultural responsibility for country whereas 29.1% of non-Rangers said they lived on country, 21.5% spent a lot of time on country and 26.5% had cultural responsibilities for country. In both Rangers and non-Rangers, there were higher proportions of participants reporting spending a lot of time on country in the Central Australian group compared to the non-Central Australian group (Table 3).

The associations between cultural variables and the wellbeing outcomes are presented in Supplementary Material Table S4.

### 3.3. Wellbeing Outcomes of Rangers Compared to Non-Rangers, by Location

#### 3.3.1. Central Australia

The first aim of the paper was to determine if associations continued to be observed among Central Australian participants. In Central Australia, the prevalence of very high life satisfaction (1.31, 1.09–1.57) and high family wellbeing (1.17, 1.01–1.36) were significantly higher in the Ranger group compared to non-Rangers (Table 4). The association between Ranger status and very high life satisfaction, and between Ranger status and high family wellbeing remained significant after adjusting for education, financial status, health condition score and health risk factor score. The association between Ranger status and higher general health were positive but not significant in Central Australia (1.06, 0.98–1.15) and did not change after controlling for education, financial status, employment, health condition score and health risk factor score. We did not observe a relationship between Ranger status and psychological wellbeing. The results concord with the earlier proof-of-concept study findings.

#### 3.3.2. Non-Central Australia

Our second aim was to examine if wellbeing results were also observable among Rangers that reside outside Central Australia. The results were not sensitive to difference in geographic location, we observed similar positive associations among Rangers and very high life satisfaction (1.29, 1.06–1.57); and Rangers and high family wellbeing (1.37, 1.14–1.65) in this group (Table 4). General health was similarly a positive association but non-significant (1.02, 0.91–1.21). Finally, we did not observe a relationship between Rangers’ status and psychological wellbeing. The associations with wellbeing were similar despite the differences in sociodemographic (Table 2), culture (Table 3) and health characteristics (Supplementary Table S3) between non-Central and Central Australian groups.

The analysis of Rangers in non-Central Australia provided the opportunity to adjust for remoteness. When remoteness was adjusted for in the analysis, the associations between Rangers and high life satisfaction (1.31, 1.08–1.60), and between Rangers and family wellbeing (1.33, 1.14–1.56) was not sensitive to remoteness (see Table 4).

### 3.4. Mediation between Ranger, Culture and Wellbeing Outcomes

The final aim of this study was to determine if Ranger status and/or factors of culture (language and connection to country) mediated the relationships observed. For this analysis, we examined the two wellbeing outcomes which had a significant association with Ranger status—family wellbeing and life satisfaction among all participants (*n* = 9691) (see Table 4).

**Figure 1:**
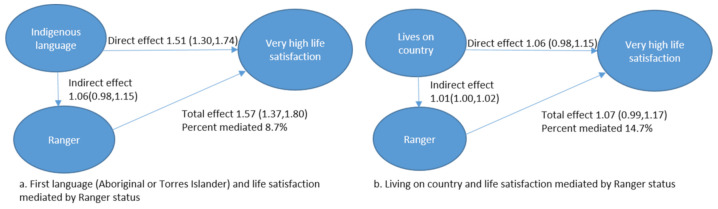
Directed Acyclic Graph showing relationships between life satisfaction and cultural factors ((**a**). first language, (**b**) living on country) mediated by Ranger.

When cultural variables (first language and living on your country) were assumed to come before Ranger employment, we found the direct effect of first language on life satisfaction was 1.51 (1.30,1.57), which increased to 1.57 (1.37,1.80) when accounting for the mediating effect of Ranger status (Figure 1a). Ranger status partially mediated the relationship between first language and very high life satisfaction (8.7%). Similarly, we found the direct effect of living on country and life satisfaction was 1.06 (0.98,1.15), which increased slightly to 1.07 (0.99,1.17) when accounting for the mediating effect of Ranger status. Ranger status partially mediated the relationships between a participant’s first language and life satisfaction (14.7%).

**Figure 2:**
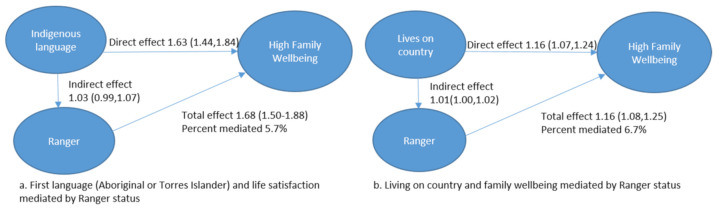
Directed Acyclic Graph showing relationships between cultural factors ((**a**). first language, (**b**). living on country) and very high life satisfaction mediated by Ranger status.

For outcomes of family wellbeing, we also observed that Ranger status partially mediated the relationships between first language and family wellbeing (5.7%), and between connection to country and family wellbeing (6.7%) (Figure 2). For both outcomes, the addition Ranger status as a mediator was positive, increasing the overall effect size between the wellbeing outcome and cultural factor.

#### 3.4.1. Cultural Factors Mediating the Association between Ranger Status and Very High Life Satisfaction

When cultural variables were considered to mediate the relationship between Ranger status and wellbeing outcomes, we found a similar positive mediation effect on wellbeing outcomes. Confidence in speaking language mediates up to 58.3% of association between Ranger status and very high life satisfaction, with the proportion mediated decreasing at each cut point (Table 5). Speaking an Aboriginal or Torres Strait Islander language mediates up to 36.4% of the association between Ranger status and very high life satisfaction, with the proportion mediated increasing at each cut point.

Maintaining a connection to country mediated the relationship between Ranger status and very high life satisfaction. Cultural responsibilities for country mediates 11.0% of the association between Ranger status and very high life satisfaction. The amount of time spent on country mediated between 50.9–71.8% of the relationship between Ranger status and very high life satisfaction.

#### 3.4.2. Cultural Factors Mediating the Association between Ranger Status and High Family Wellbeing

Speaking an Aboriginal and/or Torres Strait Islander language and confidence in speaking language mediates up to 24.2% and 28.6% respectively of the association between Ranger status and high family wellbeing, with the proportion mediated decreasing at each cut point (Table 6). Spending time on country mediates up to 52.0% of the association between Ranger status and high family wellbeing.

## 4. Discussion

This study demonstrates evidence of consistent positive associations between Ranger status and wellbeing outcomes and provides the first national level analysis of wellbeing outcomes associated with Indigenous Ranger work in Australia. Across both geographic locations, Rangers had a significantly higher prevalence of high family wellbeing and very high life satisfaction, replicating our 2017 findings [25]. Positive associations were also observed for general health, but were not statistically significant. These results strengthen evidence of associations between Ranger work and wellbeing among Aboriginal and Torres Strait Islander people and demonstrate the relationships are partially mediated by participants’ use of Aboriginal language and maintaining their connection to country. The findings support the importance of the Ranger program beyond the employment, economic and environmental benefits of Ranger work.

We previously hypothesized that higher wellbeing outcomes among Rangers could partially be explained by increased participation in cultural activities through their employment. Our findings suggest that Ranger work and cultural participation together have a reinforcing effect on wellbeing outcomes. We observed that maintaining connections to country and speaking Aboriginal and Torres Strait Islander languages positively mediates the associations between Ranger and two wellbeing outcomes. These are complex relationships and multidirectional. It is likely that participants who have a strong connection to country and speak an Indigenous language will be attracted to and selected for Ranger work. It is also likely that Ranger work reinforces cultural participation. This corroborates other research that demonstrated Ranger work reinforces culture, facilitates connection to country, expands use of language and enables Rangers to connect with Elders transmitting cultural knowledge and learning [23]. In an applied sense this means Ranger employment utilizes and facilitates the: sharing knowledge and stories of sacred sites, learning tracks of endangered animals, knowing when plants flower, and passing the responsibility to look after sites, fauna and flora between generations. Walsh and Miles (2017) highlighted that harnessing culture and traditional knowledge in Ranger work is valued by Rangers, Traditional Owners and other community members, extending potential outcomes beyond the immediate Ranger cohort [33]. The findings point to the benefits of employment practices that can dually draw on existing cultural practice and extend cultural participation and adds to the accumulating evidence of the role of culture in improved wellbeing [13,34,35].

There remains a challenge in quantitative assessments of culture in epidemiological analysis, as conceptually culture is a social phenomenon which is dynamic and explained through relationships with others. It can rarely be dichotomized among participants (as those with and without) and many questions are needed to capture different aspects, even within concept of connection to country and language. We have demonstrated in this paper the use of three different questions for both concepts of language and connection to country. In part, the construct of questions in the Mayi Kuwayu Study are intended to capture broad experiences of culture and reflect that culture should be considered as everyday practice rather than as one-off events. We suggest that measures that capture a continuum of culture may be more purposeful than either questions that ask participant’s about a point in time, such as “have you participated in an event” (as currently used by ABS Aboriginal and Torres Strait Islander Social Survey) or analysis that compares those with to those without. Additionally, questions that consider the quality of connection to language (for example, how confident are you, how important is it that speak language) and country (do you have cultural responsibilities for…) are as important as those which ask about quantity (how much time...). Our mediation analysis demonstrated that results are sensitive to various questions and cut-off points. Further work in the Mayi Kuwayu Study on the cultural measures would help to elucidate reasons for these findings.

It is highly probable that being a Ranger is a particularly important strategy for improving wellbeing outcomes among Aboriginal and Torres Strait Islander peoples who live in remote and regional areas. Remote and very remote areas have greater numbers of Ranger programs (see map [36]) and this also coincides with areas that are more socially and economically disadvantaged, with lower employment levels and higher prevalence of chronic disease among the population [37]. As such, while outcomes of very high life satisfaction and high family wellbeing continued to be observed in Rangers when remoteness area was controlled for in the analysis, we suggest that this strategy is of critical importance in the regional and remote areas in which there are limited opportunities for employment and where poorer socioeconomic conditions are seen generally. The examination of area level variables on socioeconomic or income inequality in future analysis could be considered.

An association between Ranger status and high psychological wellbeing was not found, which is also consistent with our proof-of-concept findings. The Kessler 5 and 10 instruments have been validated for use within the Aboriginal and Torres Strait Islander population [38,39]. However, this validation research similarly did not demonstrate associations between cultural identity and the social and emotional measures, including Kessler 5 [38]. Recent research has demonstrated that Aboriginal and Torres Strait Islander wellbeing is best understood within a holistic framework and that it is optimal to use measures of wellbeing that are Indigenous developed [40]. The use of mainly standard, subjective, Western-centric measures of wellbeing poses a limitation to our research.

An important strength of this research is demonstrating consistent cross-sectional trends in outcomes in Central Australian Rangers and similar results across the rest of the Mayi Kuwayu cohort. These findings provide national-level evidence demonstrating that our observed associations are not limited to small, localized samples and are replicable across time. This analysis starts to address past critiques that culture and health studies have been only been demonstrated in localized populations at one point in time (see for example Kowal 2015 [41]). Our results demonstrated replicability when controlling for differences in gender, age group, education, family financial status and remoteness. A limitation of this analysis is that there may be some differences between the Central Australia and non-Central Australia groups which could not be controlled for in our methods. This may include the different impacts of colonization, policy implementation of Ranger programs, other employment options, as well as access to health and education services which all impact on the outcomes. It is beyond the scope of the paper to identify all of the unmeasured and confounding factors. Our approach to replicate the 2017 analysis in a larger population has strengths to building the evidence, but also means that we focused on a limited number of health conditions and health risk factors, and our analysis adjusted for covariates individually rather simultaneously. The research demonstrated consistent results and this was despite a number of minor changes to survey questions between the proof-of-concept and this study (highlighted in Table 1 and Supplementary Table S1). The changes to questions were intended as improvements and enhanced the number of completed responses. Another limitation of the analysis was that it is not possible to distinguish current and past Ranger status or length of time a Ranger had been employed which is likely to influence results. Despite this, our results show the positive associations between Ranger and wellbeing regardless of how long a participant was engaged in Ranger activities. This analysis will be improved with longitudinal data, although we note that the follow-up of Rangers in Central Australia proved challenging in this wave. Only 17 Central Australian Rangers of the 43 surveyed in the proof-of-concept were re-surveyed in this wave of data collection, 26 participants were lost to follow-up, which is too small a sample for a longitudinal analysis. Longitudinal data or the addition of time of employment as a Ranger would be useful, particularly for examining the relationships between Ranger status and general health. We hypothesize that the relationship between general health and Ranger work may be stronger over time given the physical nature of Ranger work. Overall, future analysis of longitudinal data will help in determining the attribution of associations and causality. We hypothesize that the relationships are likely to be multidirectional and complex, and may require structural equation modelling, to examine multiple factors and constructs of culture concurrently.

Our research supports the call for Indigenous Ranger programs to continue to be expanded, with recent increased funding to the Working on Country program considered important for public health outcomes. From Schultz and Cairney’s perspective, “the relationship between people and their country is rarely considered in policy or service development” and “Aboriginal Community Controlled Health Services… have the capacity to provide cultural services including land management” [42]. We argue that these calls potentially disregard the historical and recent work of Land Councils to facilitate people’s access to “caring for country” opportunities. In the Northern Territory Land Councils, land management policy and programs have been facilitated through the Aboriginal Land Rights Act (NT), Indigenous Protected Area, and to lesser extent Native Title [43]. The Northern Territory may be unusual, as over 50% of the land mass is owned by Aboriginal people facilitating opportunities to manage and maintain country [44]. However, this potentially adds weight to the need for greater public sector investment in “caring for country” initiatives in other jurisdictions. Such investment has been advocated by Hunt based on her research in New South Wales, where only 1% of land is Aboriginal owned [45]. Interestingly, our results also showed that spending time on country and having cultural responsibilities for country may be just as (or potentially more) important than the permanent occupancy of land for wellbeing outcomes. Recently announced targets in the new National Partnership Agreement on Closing the Gap, obligate the Australian government and State/Territory governments to a 15% increase in the landmass and areas of sea covered “by Aboriginal and Torres Strait Islander people’s legal rights or interest” [19]. This is a worthy policy commitment, but perhaps equal consideration should also be given to program targets that provide time on country and utilize people’s cultural responsibilities to country.

While land management has not traditionally aligned with public health interventions, it could be an important addition to population health approaches aimed at improving wellbeing among Aboriginal and Torres Strait Islander peoples. The Ranger program demonstrates that there is value in better aligning public health with development approaches (and Indigenous employment options) that reinforce (or revitalize) culture. The principles demonstrated in this study show that utilizing cultural knowledge and engaging in cultural participation (using language and maintaining Aboriginal language, being on country) supports wellbeing. Applying these principles to other programs and employment could support wellbeing outcomes more broadly among Aboriginal and Torres Strait Islander people who are not Rangers. Critics have argued that the establishment of Indigenous specific employment creates a set of non-mainstream employment pathways that are inefficient and unsustainable [46,47]. With these researchers also pathologizing culture as the cause of Indigenous socioeconomic disadvantage and ill-health (see discussion by Austin-Broos [48]). We disagree with the critiques of Indigenous based employment and culture because it assumes that the non-Indigenous economic pathways are optimal and culture is a problem. Our findings support the arguments that culture strengthens wellbeing and reduces disadvantage among Aboriginal and Torres Strait Islander peoples [35,49,50]. Using and reinforcing culture and cultural knowledge in workplaces and employment has very practical benefits for Aboriginal and Torres Strait Islander people. This work supports the rights of individuals and families to make choices on where they live, their lifestyle and the levels of interaction they have with services.

## 5. Conclusions

The positive associations observed in our 2017 proof-of-concept analysis continued for Central Australia Rangers and were also found among other Rangers across Australia. Ranger work is linked to higher levels of very high life satisfaction and high family wellbeing for the Mayi Kuwayu participants. These associations are positively mediated by the cultural engagement, including the use and confidence to speak your Aboriginal language and spending time on or having responsibilities for country. Overall, our evidence supports the continuation of Working on Country program, because of the many benefits to Aboriginal Rangers and the communities where Ranger programs are established. Rangers and their communities substantiate this when they identify Ranger work as “the most important job in my community” [51]. Ranger work that draws on cultural knowledge and reinforces participation in cultural activities is not only good for land, but it is good for people. Given the strong relationship that culture has to wellbeing, determining policies that mutually acknowledge and enhance cultural knowledge and ongoing cultural participation will likely have additional benefits for the broader Aboriginal and Torres Strait Islander population.

## Figures and Tables

**Table 1:** Comparison between 2017 proof-of-concept study and current study using Mayi Kuwayu baseline data.

Study Elements	2017 Proof of Concept Study [25]	Current Study—Using Mayi Kuwayu Data
Sample size	Total sample: 203 43 Rangers: 160 non-Rangers	Central Australia participants: 102 Rangers, 494 non-Rangers, total 596.non-Central Australia participants: 164 Rangers, 8931 non-Rangers, total 9095 Total participants: 266 rangers: 9425 non-Rangers, total 9691
Geographical coverage	Central Australia	National, includes participants in all states and remoteness areas. The analysis focuses on Central Australia compared to non-Central Australia.
Recruitment process	Field-based recruitment	Postal Field-based recruitment
Outcome variables	Psychological Wellbeing (K5) Life satisfaction General Health Family wellbeing	Psychological wellbeing (K5) Life satisfaction * General Health Family wellbeing *
Adjustment variables	Financial status (income) Employment Education Health risk factor score Health condition score	Family financial status * Employment Education Health risk factor score Health condition score
Covariates	Gender	Gender, age Remoteness (non-Central Australia)
Mediation analysis	Not included	Language: first language, confidence in speaking language, intensity of speaking language Connection to country: Lives on country, spends time on country, has cultural responsibilities for country.

* Adaptations to questions from proof-of-concept survey to Mayi Kuwayu survey, see Supplementary Material Table S1.

**Table 2:** Socioeconomic and demographic characteristics of Mayi Kuwayu participants by Ranger status and geographic location.

% (*n*)	Geographic Location				Total		
	Central Australia		Non-Central Australia				
	Non-Ranger	Ranger *	Non-Ranger	Ranger *	Non-Ranger	Ranger *	Total
	*n* = 494	*n* = 102	*n* = 8931	*n* = 164	*n* = 9425	*n* = 266	*n* = 9691
Gender							
Male	32.2% (159)	61.8% (63)	37.5% (3351)	63.4% (104)	37.2% (3510)	62.8% (167)	37.9% (3677)
Female	63.0% (311)	33.3% (34)	60.1% (5364)	32.9% (54)	60.2% (5675)	33.1% (88)	59.5% (5763)
Missing	4.9% (24)	4.9% (5)	2.4% (216)	3.7% (6)	2.5% (240)	4.1% (11)	2.6% (251)
Age group							
16–34	41.3% (204)	44.1% (45)	29.4% (2629)	26.2% (43)	30.1% (2833)	33.1% (88)	30.1% (2921)
35–54	39.1% (193)	39.2% (40)	30.3% (2707)	39.0% (64)	30.8% (2900)	39.1% (104)	31.0% (3004)
<54	19.6% (97)	16.7% (17)	40.3% (3595)	34.8% (57)	39.2% (3692)	27.8% (74)	38.9% (3766)
Missing	5.3% (26)	7.3% (12)	8.5% (756)	7.3% (12)	8.3% (808)	7.1% (19)	8.4% (789)
Employment							
unemployed	56.9% (281)	19.6% (20)	46.6% (4164)	46.3% (76)	47.2% (4445)	36.1% (96)	46.9% (4541)
employed	43.1% (213)	80.4% (82)	53.4% (4767)	53.7% (88)	52.8% (4980)	63.9% (170)	53.1% (5150)
Financial Status							
Low	20.0% (99)	18.6% (19)	15.5% (1380)	18.3% (30)	15.7% (1479)	18.4% (49)	15.8% (1528)
Medium	54.7% (270)	57.8% (59)	68.6% (6131)	65.9% (108)	67.9% (6401)	62.8% (167)	67.8% (6568)
High	3.0% (15)	≤19.6% (≤20)	6.1% (549)	6.7% (11)	6.0% (564)	5.6% (15)	6.0% (579)
Missing or unsure	22.3% (110)	≤19.6% (≤20)	9.8% (871)	9.1% (15)	10.4% (981)	13.2% (35)	10.5% (1016)
Education level							
Yr 10 or less	66.6% (329)	61.8% (63)	43.4% (3880)	43.9% (72)	44.7% (4209)	50.8% (135)	44.8% (4344)
Yr 12, Trade or Tertiary	24.3% (120)	32.4% (33)	37.2% (3324)	33.5% (55)	36.5% (3444)	33.1% (88)	36.4% (3532)
University	3.8% (19)	≤4.9% (≤5)	17.5% (1560)	21.3% (35)	16.8% (1579)	14.7% (39)	16.7% (1618)
Missing	5.3% (26)	≤4.9% (≤5)	1.9% (167)	1.2% (2)	2.0% (193)	1.5% (4)	2.0% (197)
Remoteness Area							
major cities	0	0	44.3% (3958)	22.0% (36)	42.0% (3958)	13.5% (36)	41.2% (3994)
inner regional	0	0	30.7% (2741)	26.8% (44)	29.1% (2741)	16.5% (44)	28.7% (2785)
outer regional	1.8% (9)	≤4.9% (≤5)	19.4% (1734)	31.1% (51)	18.5% (1743)	19.5% (52)	18.5% (1795)
remote	29.8% (147)	11.8% (12)	1.6% (140)	8.5% (14)	3.0% (287)	9.8% (26)	3.2% (313)
very remote	65.4% (323)	84.3% (86)	2.5% (224)	7.9% (13)	5.8% (547)	37.2% (99)	6.7% (646)
missing	3.0% (15)	≤4.9% (≤5)	1.5% (134)	3.7% (6)	1.6%(149)	3.4% (9)	1.6% (158)

* Ranger included anyone who was formerly or is currently a Ranger.

**Table 3:** Cultural characteristics of Rangers and non-Rangers, by geographic location.

	Geographic Location				Total		
	Central Australia Ranger Status		Non-Central Australia Ranger Status				
	Non-Ranger	Ranger *	Non-Ranger	Ranger *	Non-Ranger	Ranger *	Total
	*n* = 494	*n* = 102	*n* = 8931	*n* = 164	*n* = 9425	*n* = 266	*n* = 9691
What is your first language?							
English or other	36.2% (179)	23.5% (24)	93.5% (8353)	81.1% (133)	90.5% (8532)	59.0% (157)	89.7% (8689)
Aboriginal/Torres Strait Islander	56.1% (277)	61.8% (63)	2.8% (251)	8.5% (14)	5.6% (528)	28.9% (77)	6.2% (605)
Missing	7.7% (38)	14.7% (15)	3.7% (327)	10.4% (17)	3.9% (365)	12.0% (32)	4.1% (397)
Do you speak any Aboriginal/Torres Strait Islander language?							
No	17.6% (87)	6.9% (7)	64.0% (5713)	25.6% (42)	61.5% (5800)	18.4% (49)	60.4% (5849)
Yes, a little bit	24.3% (120)	22.5% (23)	27.8% (2487)	50.6% (83)	27.7% (2607)	39.8% (106)	28.0% (2713)
Yes, a fair bit	9.1% (45)	6.9% (7)	2.8% (253)	14.6% (24)	3.2% (298)	11.7% (31)	3.4% (329)
Yes, a lot	41.9% (207)	55.9% (57)	1.4% (123)	5.5% (9)	3.5% (330)	24.8% (66)	4.1% (396)
Missing	7.1% (35)	7.8% (8)	4.0% (355)	3.7% (6)	4.1% (390)	5.3% (14)	4.2% (404)
I am confident in speaking language.							
Want to but can’t	6.9% (34)	2.0% (2)	26.6% (2377)	16.5% (27)	25.6% (2411)	10.9% (29)	25.2% (2440)
Not at all	7.3% (36)	4.9% (5)	27.7% (2478)	9.8% (16)	26.7% (2514)	7.9% (21)	26.2% (2535)
A little bit	16.8% (83)	17.6% (18)	15.4% (1377)	34.1% (56)	15.5% (1460)	27.8% (74)	15.8% (1534)
A fair bit	7.3% (36)	6.9% (7)	4.8% (429)	13.4% (22)	4.9% (465)	10.9% (29)	5.1% (494)
A lot	53.2% (263)	63.7% (65)	3.9% (350)	13.4% (22)	6.5% (613)	32.7% (87)	7.2% (700)
Unsure	3.0% (15)	1.0% (1)	8.2% (730)	3.0% (5)	7.9% (745)	2.3% (6)	7.7% (751)
Missing	5.5% (27)	3.9% (4)	13.3% (1190)	9.8% (16)	12.9% (1217)	7.5% (20)	12.8% (1237)
Do you currently live on your country/Island?							
no/unsure	51.6% (255)	41.2% (42)	68.2% (6094)	51.2% (84)	67.4% (6349)	47.4% (126)	66.8% (6475)
yes	43.9% (217)	52.9% (54)	28.2% (2521)	43.9% (72)	29.1% (2738)	47.4% (126)	29.6% (2864)
Missing	4.5% (22)	5.9% (6)	3.5% (316)	4.9% (8)	3.6% (338)	5.3% (14)	3.6% (352)
How much time do you spend on country?							
Want to but can’t	4.0% (20)	0.0% (0)	16.2% (1450)	11.0% (18)	15.6% (1470)	6.8% (18)	15.4% (1488)
Not at all	9.3% (46)	0.0% (0)	27.5% (2454)	6.1% (10)	26.5% (2500)	3.8% (10)	25.9% (2510)
A little bit	19.6% (97)	5.9% (6)	28.1% (2510)	25.6% (42)	27.7% (2607)	18.0% (48)	27.4% (2655)
A fair bit	16.6% (82)	22.5% (23)	11.1% (989)	22.6% (37)	11.4% (1071)	22.6% (60)	11.7% (1131)
A lot	42.5% (210)	65.7% (67)	8.3% (743)	29.3% (48)	10.1% (953)	43.2% (115)	11.0% (1068)
Missing	7.9% (39)	5.9% (6)	8.8% (785)	5.5% (9)	8.7% (824)	5.6% (15)	8.7% (839)
Do you have cultural responsibility for country?							
no cultural responsibilities for country	31.8% (157)	17.6% (18)	75.8% (6767)	42.1% (69)	73.5% (6924)	32.7% (87)	72.3% (7011)
cultural responsibilities for country	68.2% (337)	82.4% (84)	24.2% (2164)	57.9% (95)	26.5% (2501)	67.3% (179)	27.7% (2680)

* Ranger included anyone who was formerly or is currently a Ranger.

**Table 4:** Associations between Ranger status and wellbeing of Rangers and non-Rangers, for Central Australia and non-Central Australia.

Non-Ranger (Reference Group) 1.0	Central Australia			Non-Central Australia		
	*n*	PR	95% CI	*n*	PR	95% CI
**Very high life satisfaction**	**556**	**1.31**	**1.09,1.57**	**8811**	**1.29**	**1.06,1.57**
adjusted for education	556	1.32	1.09,1.58	8811	1.28	1.05,1.56
adjusted for financial status	556	1.28	1.07,1.55	8811	1.26	1.05,1.50
adjusted for employment	556	1.35	1.13,1.62	8811	1.32	1.08,1.60
adjusted for health condition score	556	1.29	1.07,1.55	8811	1.34	1.10,1.63
adjusted for risk factor score	556	1.29	1.07,1.55	8811	1.33	1.10,1.61
adjusted for remoteness				8811	1.31	1.08,1.60
**Very good general health**	**572**	**1.06**	**0.98,1.15**	**8868**	**1.02**	**0.91,1.13**
adjusted for education	554 *	1.07	0.98,1.16	8868	0.99	0.89,1.09
adjusted for financial status	553 *	1.05	0.97,1.15	8868	0.98	0.89,1.07
adjusted for employment	572	1.06	0.97,1.16	8868	1.01	0.92,1.11
adjusted for health condition score	572	1.06	0.98,1.15	8868	1.04	0.94,1.14
adjusted for risk factor score	528 *	1.07	0.98,1.17	8868	1.04	0.95,1.15
adjusted for remoteness				8868	1.02	0.92,1.13
**High psychological wellbeing**	**510**	**0.87**	**0.66,1.15**	**8240**	**1.05**	**0.92,1.21**
adjusted for education	510	0.95	0.71,1.27	8240	1.08	0.94,1.23
adjusted for financial status	510	0.96	0.72,1.28	8240	1.02	0.90,1.16
adjusted for employment	510	0.91	0.68,1.22	8240	1.07	0.94,1.22
adjusted for health condition score	510	0.95	0.72,1.26	8240	1.05	0.89,1.24
adjusted for risk factor score	510	0.96	0.72,1.27	8240	1.05	0.92,1.20
adjusted for remoteness				8240	1.09	0.95,1.25
**High family wellbeing**	**538**	**1.17**	**1.01,1.36**	**8465**	**1.37**	**1.14,1.65**
adjusted for education	538	1.35	1.15,1.58	8465	1.36	1.17,1.59
adjusted for financial status	538	1.18	1.01,1.38	8465	1.37	1.18,1.58
adjusted for employment	538	1.23	1.05,1.43	8465	1.38	1.18,1.61
adjusted for health condition score	538	1.20	1.03,1.39	8465	1.38	1.18,1.62
adjusted for risk factor score	538	1.21	1.04,1.41	8465	1.39	1.19,1.63
adjusted for remoteness				8465	1.33	1.14,1.56

The **bold result** shows the main outcome adjusted for gender and age, each covariate is separately adjusted for and all multivariate analysis adjusts for gender and age (and included missing category on control variables). * Outcomes in general health in Central Australia were only adjusted for gender.

**Table 5:** Mediation analysis of cultural variables on relationship between Ranger status and very high life satisfaction.

Mediator	Natural Direct Effect *	Natural Indirect Effect *	Total Effect *	% Mediated
	PR (95% CI)	PR (95% CI)	PR (95% CI)	
*Do you speak any Aboriginal/Torres Strait Islander words/language?*				
≥A little bit	1.39 (1.06,1.85)	1.06 (0.89,1.36)	1.54 (1.27,1.85)	13.5%
≥A fair bit	1.35 (0.99,1.83)	1.13 (0.98,1.30)	1.54 (1.27,1.88)	28.3%
A lot	1.32 (1.04,1.66)	1.17 (1.02,1.33)	1.54 (1.27,1.88)	36.4%
*Are you confident in speaking your Aboriginal language?*				
≥A little bit	1.22 (0.92,1.62)	1.32 (1.04,1.66)	1.61 (1.32,1.96)	58.3%
≥A fair bit	1.26 (0.97,1.62)	1.29 (1.07,1.46)	1.62 (1.32,1.97)	47.9%
A lot	1.32(1.04,1.68)	1.22 (1.04,1.67)	1.61 (1.32,1.96)	41.8%
*Has cultural responsibilities for country?*				
Yes	1.48 (1.14,1.90)	1.05 (0.89,1.25)	1.56 (1.29,1.88)	11.0%
*How much time do you spend on country?*				
≥A little bit	1.24 (0.94,1.95)	1.25 (1.02,1.51)	1.55 (1.27,1.88)	50.9%
≥A fair bit	1.11 (0.88,1.50)	1.38 (1.11,1.72)	1.54(1.27,1.88)	74.6%
A lot	1.12 (0.87,1.46)	1.37 (1.14,2.12)	1.55(1.28,1.88)	71.8%

* Analysis adjusted for age, gender, and interaction between cultural variable and exposure.

**Table 6:** Mediation analysis of cultural variables on relationship between Ranger status and high family wellbeing.

Mediator	Natural Direct Effect *	Natural Indirect Effect *	Total Effect *	% Mediated
	PR (95% CI)	PR (95% CI)	PR (95% CI)	
*Do you speak any Aboriginal/Torres Strait Islander words/language?*				
≥A little bit	1.39 (1.01,1.59)	1.11 (0.91,1.37)	1.54 (1.29,1.84)	24.2%
≥A fair bit	1.37 (1.11,1.69)	1.07 (0.96,1.23)	1.54(1.29,1.84)	15.7%
A lot	1.40 (1.16,1.71)	1.06 (0.96,1.17)	1.54 (1.29,1.84)	13.5%
*Are you confident in speaking your Aboriginal language?*				
≥A little bit	1.39 (1.06,1.81)	1.14 (0.93,1.40)	1.58 (1.32,1.88)	28.6%
≥A fair bit	1.46 (1.17,1.82)	1.08 (0.94,1.23)	1.58 (1.32,1.88)	16.8%
A lot	1.49 (1.21,1.83)	1.06 (0.95,1.18)	1.58 (1.32,1.88)	12.7%
*Has cultural responsibilities for country?*				
Yes	1.35 (1.06,1.71)	1.14 (0.98,1.35)	1.55 (1.30,1.84)	29.9%
*How much time do you spend on country?*				
≥A little bit	1.40 (1.15,1.67)	1.10 (0.92,1.33)	1.56 (1.30,1.86)	21.4%
≥A fair bit	1.24 (0.95,1.60)	1.26 (1.04,1.52)	1.56 (1.30,1.86)	52.0%
A lot	1.38 (1.10,1.71)	1.13 (0.99,1.29)	1.56 (1.30,1.87)	27.5%

* Analysis adjusted for age, gender, and interaction between cultural variable and exposure.

## Data Availability

The Mayi Kuwayu dataset is governed by a committee of Aboriginal and Torres Strait Islander leaders. All request to access data need are subject to their approval and review.

## Supplementary Materials

The following are available online at https://www.mdpi.com/1660-4601/18/6/3053/s1, Table S1: Changes in Mayi Kuwayu survey questions from proof-of-concept study to baseline survey; Table S2: Postcodes used to determine geographic locations: Central Australia and non-Central Australia; Table S3: Health conditions and health risk factors of Rangers and non-Rangers by geographic location; Table S4: Univariate analysis (prevalence rate ratio) of the relationship between cultural factors and wellbeing outcome.
