# Supplementary material for: Examining the Associations between Indigenous Rangers, Culture and Wellbeing in Australia, 2018–2020
Source: Int J Environ Res Public Health. 2021 Mar 16;18(6):3053. doi: 10.3390/ijerph18063053 (PMC8002247; doi:10.3390/ijerph18063053)
Supplement: Supplementary file 1 [file ijerph-18-03053-s001.pdf]

Supplementary Materials:

Table S1. Changes in Mayi Kuwayu survey questions from proof-of-concept study to baseline survey.

Question area	Question in the proof-of-concept study	Question in the Mayi Kuwayu baseline survey
Financial status	Q: Given your current needs and financial responsibilities, indicate if you are: very poor, poor, just getting along, reasonably comfortable, very comfortable or prosperous'.<br><br>Recoding: Responses were categorized as low financial status (very poor, poor or just getting along) or high financial status (reasonably comfortable, very comfortable or prosperous).	Q: "Which words best describe your family's money situation"? Responses included: "we have a lot of savings", "we have some savings", "we have just enough to get us to the next payday", "we run out of money before payday" or "we are spending more than we get".<br><br>Recoding: Responses were recoded to: high (included participants who selected "we have a lot of savings"), medium (included participants who selected "we have some savings" "we have just enough to get us to the next payday"), low (included participants who selected "we run out of money before payday" or "we are spending more than we get"), and missing (included responses of unsure or missing).
Family wellbeing	Q: A modified Western Australian Aboriginal Child Health Survey family functioning scale<br><br>Answer: Response options of 'not at all' (1) to 'very much' (5).<br><br>Recoding: Responses were summed (range: 9–45), and participants were categorized as having low/moderate (score 9–36) or high family wellbeing (score 37–45).	Q: A modified Western Australian Aboriginal Child Health Survey family functioning scale.<br><br>Answer: Response options included not at all, a little bit, a fair bit, a lot, unsure<br><br>Recoding: Responses were summed (range: 0–36), and participants were categorised as having low/moderate (score 0–29) or high family wellbeing (score 30–36).
Life Satisfaction	Q: How satisfied are you with your life as a whole? A: A scale from 0 (completely dissatisfied) to 10 (completely satisfied).<br><br>Recoding: Scores were categorized as low to high life satisfaction (score 0–8) or very high life satisfaction (score 9–10).	Q: How satisfied are you with your life? A: A lot, A fair bit, a little bit, Not at all.<br><br>Recoding: Categorised as low to high life satisfaction (a little bit, not at all, a fair bit) or very high life satisfaction (a lot).

Table S2. Postcodes used to determine geographic locations: Central Australia and non-Central Australia.

Geographic location	Postcodes
Central Australia	0870, 0871, 0872, 0861, 0862, 0852, 4825, 5723, 6765, 6770, 6753. 6430, 6433, 6431
non-Central Australia	All remaining participants, including those with missing postcode.

*Central Australia is often colloquially termed as the southern part of the Northern Territory. In our paper, Central Australia includes not only the southern part of the NT, but is a larger geographic zone that overlaps state and territory boundary of South Australia, Western Australian and Northern Territory. It also extends to the Barkly region, and includes Lajamanu and Dagaragu communities, encompassing the service area of the Central Land Council. This wider region of Central Australia ensures that related and closely associated (through for example, kinship and language groups) Aboriginal and Torres Strait Islander groups are included in our analysis.

Table S3. Health conditions and health risk factors of Rangers and non-Rangers by geographic location.

**% (n)**	Geographic location				TOTAL		
	Central Australia		non-Central Australia				
	non-Ranger	Ranger*	non-Ranger	Ranger*	non-Ranger	Ranger*	Total
	N=494	N=102	N=8931	N=164	N=9425	N=266	N=9691
			Heart disease				
no	91.3% (451)	91.2% (93)	88.8% (7933)	86.6% (142)	89.0%(8384)	88.3%(235)	88.9%(8619)
yes	8.7% (43)	8.8% (9)	11.2% (998)	13.4% (22)	11.0%(1041)	11.7%(31)	11.1%(1072)
Stroke							
no	**	**	97.3% (8687)	95.1% (156)	97.4%(9180)	97.0%(258)	97.4%(9438)

yes	**	**	2.7% (244)	4.9% (8)	2.6%(245)	3.0%(8)	2.6%(253)
Diabetes							
no	80.4% (397)	76.5% (78)	83.0% (7409)	79.9% (131)	95.8%(9027)	97.4%(259)	95.8%(9286)
yes	19.6% (97)	23.5% (24)	17.0% (1522)	20.1% (33)	4.2%(398)	2.6%(7)	4.2%(405)
Health Condition score							
No conditions	75.7% (374)	72.5% (74)	75.1% (6704)	72.6% (119)	75.1%(7078)	72.6%(193)	75.0%(7271)
1 or more	24.3% (120)	27.5% (28)	24.9% (2227)	27.4% (45)	15.1%(1419)	27.4%(73)	25.0%(2420)
High blood pressure							
no	82.2% (406)	77.5% (79)	67.2% (6006)	64.6% (106)	68.0%(6412)	69.5%(185)	68.1%(6597)
yes	17.8% (88)	22.5% (23)	32.8% (2925)	35.4% (58)	32.0%(3013)	30.5%(81)	31.9%(3094)
High cholesterol							
no	89.5% (442)	93.1% (95)	72.0% (6426)	73.2% (120)	72.9%(6868)	80.8%(215)	73.1%(7083)
yes	10.5% (52)	6.9% (7)	28.0% (2505)	26.8% (44)	27.1%(2557)	19.2%(51)	26.9%(2608)
Smoking							
Current smoker	44.3% (219)	47.1% (48)	24.4% (2176)	35.4% (58)	25.4%(2395)	39.8%(106)	25.8%(2501)
Never smoker	8.7% (43)	15.7% (16)	33.7% (3008)	29.3% (48)	32.4%(3051)	24.1%(64)	32.1%(3115)
Past smoker	41.9% (207)	31.4% (32)	39.4% (3522)	33.5% (55)	39.6%(3729)	32.7%(87)	39.4%(3816)
Missing	5.1% (25)	5.9% (6)	2.5% (225)	1.8% (3)	2.7%(250)	3.4%(9)	2.7%(259)
Risk factor score							
No risk factors	37.2% (184)	29.4% (30)	40.3% (3597)	31.1% (51)	40.1%(3781)	30.5%(81)	39.9%(3862)
1 or more	57.7% (285)	64.7% (66)	57.2% (5109)	67.1% (110)	57.2%(5394)	66.2%(176)	57.5%(5570)
Missing	5.1% (25)	5.9% (6)	2.5% (225)	1.8% (3)	2.7%(250)	3.4%(9)	2.7%(259)

**Not reported due to small cell count <5. *Ranger included anyone who was formerly or is currently a Ranger.

Table S4: Univariate analysis (prevalence rate ratio) of the relationship between cultural factors and wellbeing outcome.

univariate analysis		**Very high life satisfaction**	**High family wellbeing**	**Good general health**	**High psychological wellbeing**
First language	English/Other	1.0 (base)	1.0 (base)	1.0 (base)	1.0 (base)
	Aboriginal or Torres Strait Islander	**1.54 (1.40,1.69)**	**1.68(1.57,1.80)**	**1.20(1.15,1.25)**	1.05 (0.96,1.15)
	Missing	1.09 (0.94,1.28)	**1.15 (1.01,1.31)**	0.94 (0.87,1.02)	0.85 (0.74,0.98)
Confident to speak	Not at all	1.0 (base)	1.0 (base)	1.0 (base)	1.0 (base)
	A little bit	0.85 (0.77, 0.94)	**1.27 (1.17,1.37)**	1.01 (0.97,1.05)	1.06 (0.99,1.13)
	A fair bit	1.08 (0.95,1.24)	**1.47(1.32,1.62)**	**1.13 (1.06,1.20)**	**1.11 (1.00,1.23)**
	A lot	**1.52 (1.39,1.67)**	**1.86 (1.72,2.00)**	**1.19 (1.14,1.25)**	1.05 (0.96,1.15)
	Want to but can't	0.77 (0.70,0.84)	0.92 (0.85,0.99)	1.00(0.97,1.04)	1.03 (0.97,1.10)
	Unsure	0.88 (0.77,1.0)	0.92(0.82,1.03)	(0.87,0.98)	1.00 (0.92,1.10)
	Missing	1.01 (0.92, 1.12)	**1.17 (1.06,1.28)**	0.93 (0.89,0.98)	0.95 (0.87,1.03)
Speaks an Aboriginal or Torres Strait Islander language	No	1.0 (base)	1.0 (base)	1.0 (base)	1.0 (base)
	yes, a little bit	0.91 (0.85,0.98)	**1.30 (1.23,1.37)**	1.02 (0.99,1.05)	0.91 (.85,0.98)
	Yes, a fair bit	1.04 (0.88, 1.23)	**1.67 (1.51,1.85)**	**1.11 (1.04,1.18)**	**1.04 (0.89,1.23)**
	Yes, a lot	**1.75 (1.58, 1.93)**	**2.01 (1.85,2.17)**	**1.26 (1.20,1.32)**	**1.75 (1.58,1.93)**
	Missing	0.96 (0.81,1.13)	**1.33 (1.17,1.51)**	0.96 (0.89,1.04)	0.96(0.80,1.13)
Lives on country	No	1.0 (base)	1.0 (base)	1.0 (base)	1.0 (base)
	Yes	**1.05 (1.0,1.13)**	**1.12 (1.06,1.18)**	0.95 (0.90,1.00)	1.0 (0.97,1.03)
	Unsure	0.83 (0.72,0.94)	0.73 (0.64,0.83)	0.95 (0.87,1.04)	0.93 (0.87,0.99)
	Missing	1.06 (0.90,1.25)	1.00 (0.86,1.17)	0.91 (0.79,1.05)	0.90 (0.82,0.98)
Cultural responsibilities for country	No	1.0 (base)	1.0 (base)	1.0 (base)	1.0 (base)
	Yes (includes mothers, fathers, other)	**1.08 (1.01,1.16)**	**1.32(1.24,1.39)**	0.96 (0.93,0.99)	0.98(0.92,1.02)
	Unsure	0.79 (0.72,0.86)	0.80 (0.73,0.86)	0.88 (0.84,0.91)	1.0(0.94,1.06)
	Missing (unselected and missing)	0.94 (0.81,1.09)	**1.22 (1.08,1.37)**	0.94(0.87,1.00)	0.96(0.85,1.08)
Spends time on country	Not at all	1.0 (base)	1.0 (base)	1.0 (base)	1.0 (base)
	A little bit	0.84 (0.78,0.92)	**1.18 (1.09,1.27)**	1.02 (0.99,1.06)	**1.09 (1.03,1.16)**
	A fair bit	0.91 (0.82, 1.00)	**1.51 (1.39,1.63)**	1.03 (0.98,1.08)	1.05 (0.97,1.31)
	A lot	**1.30 (1.19, 1.42)**	**1.74 (1.61,1.88)**	**1.07 (1.02,1.12)**	1.03 (0.95,1.11)
	Want to but can't	0.70 (0.64,0.79)	0.89 (0.81,0.98)	0.92 (0.88,0.96)	0.99 (0.93,1.07)
	Missing	0.94 (0.83,1.06)	**1.16 (1.04,1.31)**	0.88 (0.82,0.93)	0.97 (0.88,1.07)
